# Development and validation of Simulation Scenario Quality Instrument (SSQI)

**DOI:** 10.1186/s12909-023-04935-5

**Published:** 2023-12-19

**Authors:** Gadah Mujlli, Abdulmajeed Al-Ghosen, Rola Alrabah, Fadi Munshi, Burhanettin Ozdemir

**Affiliations:** 1 Simulation and Skills Development Center, Health Affairs, Princess Nourah Bint Abdulrahman University, Riyadh, Saudi Arabia; 2 King Abdullah bin Abdulaziz University Hospital, Health Affairs, Princess Nourah Bint Abdulrahman University, Riyadh, Saudi Arabia; 3 King Salman Global Academy for Arabic Language, Riyadh, Saudi Arabia; 4 Abdulrahmans’s Oasis, The Child Life Foundation, Riyadh, Saudi Arabia; 5 Department of Mathematics and Sciences, Prince Sultan University, Riyadh, Saudi Arabia

**Keywords:** Healthcare simulation scenarios, Medical education, Simulation scenarios. Assessment instrument

## Abstract

**Background:**

Due to the unmet need for valid instruments that evaluate critical components of simulation scenarios, this research aimed to develop and validate an instrument that measures the quality of healthcare simulation scenarios.

**Methods:**

A sequential transformative mixed-method research design was used to conduct the study. The development and validation of the instrument involved two phases: the qualitative phase, which included defining the instrument's theoretical background and instrument construction, followed by the quantitative phase, where the instrument was piloted and validated. The qualitative study included 17 healthcare simulation experts, where three focus group was conducted, and the first version of the instrument was constructed based on the focus group analysis and the theoretical framework constructed using the literature review. During the quantitative phase, the instrument’s quantitative piloting included 125 healthcare simulation scenarios; then, the instrument went through construct validity and reliability testing.

**Results:**

Content experts confirmed the theoretical model and instrument framework. The average item content validity index (I-CVI) scores and the average of the I-CVI scores (S-CVI/Ave) for all items on the scale or the average proportion relevance judged by all experts was 0.87. The conformity factor analysis results showed a good fit for the proposed 10-factor model (CFI (the comparative fit index) = 0.998, Tucker-Lewis index = 0.998, Root mean square error of approximation (RMSEA) = 0.061. The final instrument included ten domains: 1. Learning objectives, 2. Target group, 3. Culture, 4. Scenario case, 5. Scenario narrative briefing, 6. Scenario complexity, 7. Scenario flow, 8. Fidelity, 9. Debriefing, and 10. Assessment. The SSQI included 44 items that are rated on a 3-point scale (Meets Expectations = (2), Needs Improvement, (1), Inadequate (0)).

**Conclusion:**

This validated and reliable instrument will be helpful to healthcare educators and simulation experts who want to develop simulation-based training scenarios and ensure the quality of written scenarios.

**Supplementary Information:**

The online version contains supplementary material available at 10.1186/s12909-023-04935-5.

## Background

Simulation-based training (SBT) in education was successfully implemented in aviation and the military. Now, it is used in healthcare to improve patient care and safety [1–3]. In the last 20 years, simulation in healthcare education has been used in healthcare education increased [4] Several studies have reported positive outcomes on healthcare students' and learners' knowledge and skills [2, 5, 6] The success of SBT depends on the careful and robust development of simulation scenarios based on critical needs and assessment instruments used to guide the delivery of specific debriefing [7, 8].

Simulated-Based Learning (SBL) Experience in healthcare education is defined as “An array of structured activities that represent actual or potential situations in education and practice. These activities allow participants to develop or enhance their knowledge, skills, and attitudes, or to analyze and respond to realistic situations in a simulated environment” [9, 10]. SBL is structured based on needs assessment to identify learning objectives and outcomes needed for the learners.

**Fig. 1:**
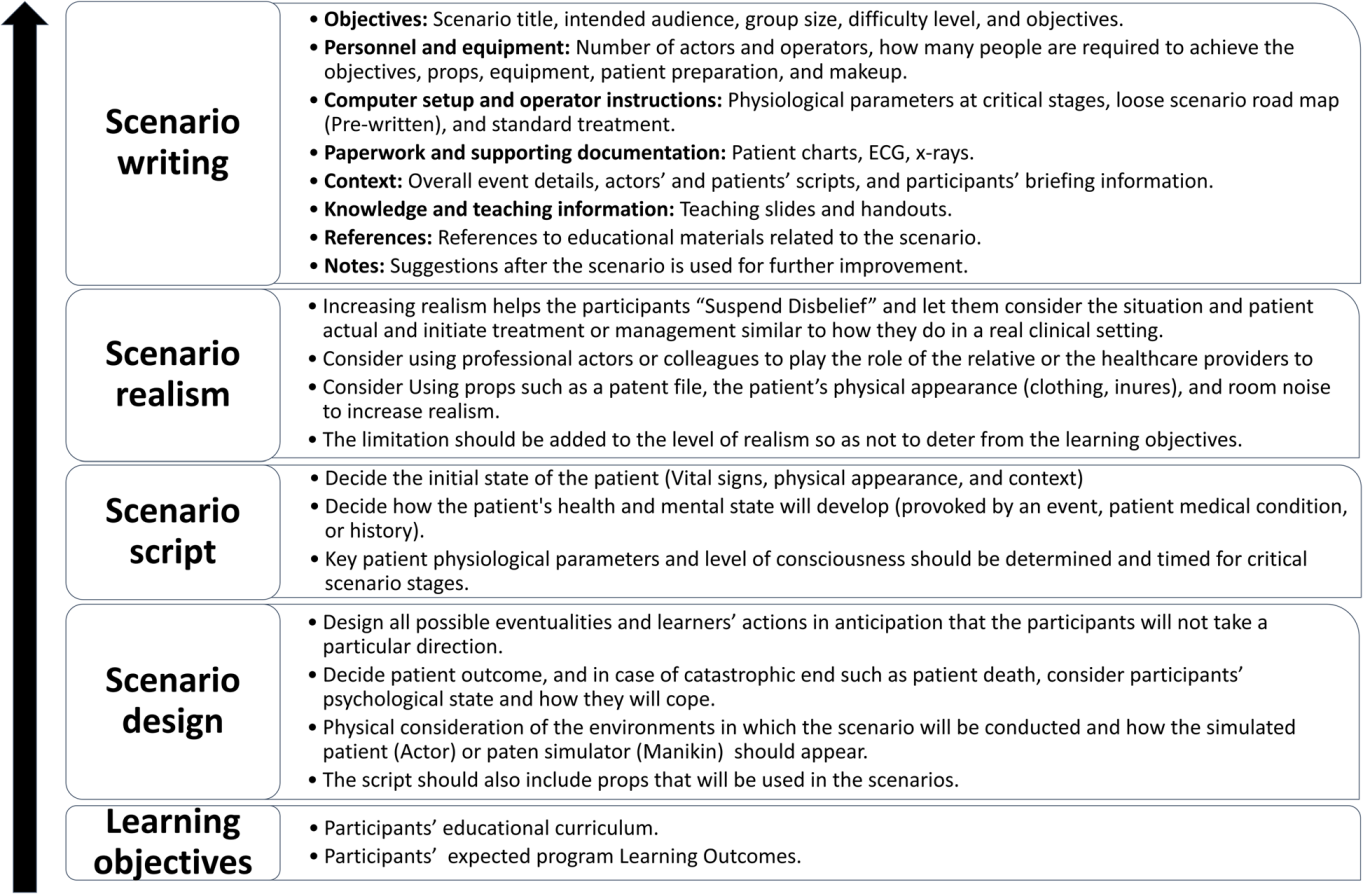
Stages of healthcare simulation scenario writing. The figure is designed based on the information provided in Alinier (2011) and Seropain’s work (2003) [1, 12]

Healthcare simulation scenarios are defined as “modeled on real-life situations that often include a sequence of learning activities that involve complex decision making, problem-solving strategies, intelligent reasoning, and other complex cognitive skills” [11]. The healthcare simulation scenarios usually include the goals, learning objectives, debriefing, scenario narrative, description of the clinical simulation encounter, staff requirements and instructions, simulation theater set up, simulation modality and operation, scenario props, and instructions for standardized patients [1]. Figure 1 identifies the significant steps and stages required to be followed to write a healthcare simulation scenario based on Alinier (2011) and Seropain's work (2003) [1, 12].

Any simulation scenario utilized for education is expected to be evidence-based in design and high quality [3, 13]. There are resources and templates published and available online to assist educators in healthcare education in developing and writing simulation scenarios [1, 13–18].

Ensuring the quality of simulation scenarios is difficult as several elements affect the simulation experience [19]. Based on the scenario development stages and steps mentioned in Fig. 1, rigorous and professional training of simulation educators and simulationists is required to ensure that they can develop and implement high-quality simulation scenarios and curriculums [20]. However, those training modules and programs are not monitored by an accreditation entity; their outcomes are their assessment, and methods are rarely reported [21]. Limited instruments evaluate some aspects of the simulation experience during conduction, namely debriefing and feedback; only one validated instrument evaluates the simulation scenario components and scenario design named “Simulation Scenario Evaluation Tool (SSET)” [22, 23]. The instrument was recently developed using the Delphi-modified method and focused on defining expectations for developing quality scenarios [22]. This instrument was developed based on the available literature and a subsequent review of six published simulation scenario templates. The instrument consists of six components that determine scenario quality that is rated based on a corresponding anchor and scale: learning objectives, critical context/scenario overview, critical actions, patient states, scenario materials and resources, and a debriefing plan [22].

This instrument is considered the first to evaluate the quality of simulation scenarios; however, the authors reported some limitations in their study [22]. It included a limited number of participants during the first and second rounds of the survey. Selection bias was identified as a limitation, and the partial response in the second survey round might have affected the analysis of certain items [22, 24]. Due to the unmet need for valid instruments that evaluate critical components of simulation scenarios, developing instruments that measure and assess the quality of the components of the healthcare simulation scenarios is vital [22]. This research aimed to develop and validate an instrument that measures the quality of the components included in the healthcare simulation scenarios.

## Methods

A sequential transformative mixed-method approach was used to develop and validate an instrument that measures the quality of healthcare simulation scenarios. The study followed two phases to create and validate the instrument: the quantitative phase, and the qualitative phase. The method of development and validation was adapted from Benson and Florence's work [25].

### Phase I: qualitative phase

Phase I is the qualitative phase of the development of the instrument. It involves two steps: the first step is planning and developing the theoretical background, and the second is instrument construction. The instrument aim, domains, and framework were defined and established in the first step based on a literature review. An extensive review of available literature discussed or reported the following two areas: the first area was the quality evaluation or assessment instruments of simulation scenarios, and the second was health simulation scenarios guidelines.

After conducting the literature review and critically appraising the evidence by two reviewers from the research team, all constructs, domains, and operational definitions of components that define the quality of simulation scenarios were summarized in overarching domains and subdomains to set up a framework for the instrument. Keywords used for research were healthcare simulation scenario, quality healthcare simulation scenario, simulation scenario guidelines, simulation scenario quality, and simulation scenario procedure. The databases included in the literature review were Cochrane Library, PubMed, Medline, Joanna Briggs Institute EBP Database, and Web of Science Core Collection.

Results of the literature review were also used to construct the script of the focus groups that included experienced simulation educators [25]. The focus group was conducted to discuss the proposed framework and investigate new themes determining the quality of healthcare simulation scenarios. Focus groups were recorded, the recordings were analyzed, and themes and concepts were established and combined with the literature review findings to finalize the instrument's framework.

The second step involved writing the instrument's items based on the established framework in the first step. After writing the items and reviewing the instrument with the research team, the instrument's content validity was determined by healthcare simulation experts. Five experts were included in the content validity process [26]. Content and face validation of the instrument was done by providing a copy to the experts. They evaluated whether the instrument accurately assessed the quality of healthcare simulation scenarios and provided feedback on each item in the instrument. The last step was revising the instrument and developing new items based on the expert's validation report.

### Phase (II): quantitative phase

The second phase included two steps: the first step was instrument piloting and the second was instrument validation. The instrument was piloted among healthcare simulation educators. The instrument was sent to them in a hard copy and an online survey with instructions to use scenarios included in the scenario library of the Simulation and Skills Development Center (SSDC) at Princess Nourah University. The scenarios in the library target different healthcare specialties. 129 scenarios were evaluated using the instrument in the pilot stage. Those scenarios were previously piloted in SSDC and archived in the library afterward. The educators included in the piloting were clinical simulation educators with experience writing and conducting health simulation scenarios. Educators with more than one year of involvement in simulation activities or who underwent training in writing health simulation scenarios have been involved in simulation activities and are staff or faculty with an educational training background.

After that, during the instrument validation step, the instrument underwent exploratory and conformity factor analysis to identify the underlying components and factors. The items that pointed to the same dimensions should have loaded into the same factors. The internal consistency of factors was checked using Cronbach's alpha coefficient. Additionally, the correlation between questions that load on the same factor was examined to ensure the instrument's answers were consistent. The reliability and validity tests results and the qualitative analysis of participants' feedback were used to determine if the instrument's items should be revised, deleted, or reduced. Changes were made to the evaluated dimensions of simulation scenarios based on these results and the theoretical background formulated in phase I. Following these revisions, the final content of the instruments for evaluating healthcare simulation scenarios was formulated and finalized.

### Statistical analysis

The qualitative analysis method of the results of the focus groups was “Constant comparison analysis” [27]. Constant comparison analysis is characterized by three stages: In the first stage (open coding), the data are chunked into small units, and the researcher attaches a code to each unit. These codes are grouped into categories during the second stage (axial coding). Finally, in the third stage (selective coding), the researcher develops themes that express the content of each group based on the categories and codes in the first and second stages [27]. Structural validity of the instrument was done using factor and confirmatory factor analysis, and Cronbach's alpha coefficients were calculated to measure the instrument's reliability. For content validity, after summarizing the reviewers’ comments, the item content validity index (I-CVI) was calculated for each item. I-CVI is defined as the proportion of content experts giving the item a relevance rating of 3 or 4 based on the following formula (I-CVI = (agreed item)/ (number of experts). An item with CVI below 0.8 was deleted. Additionally, the average of the I-CVI scores (S-CVI/Ave) for all items on the scale or the average of proportion relevance judged by all experts was calculated by this formula (S-CVI/Ave = (sum of I-CVI scores)/ (number of items)). The construct validity was investigated with three different confirmatory factor analysis (CFA) models: a one-factor model, a ten-factor model, and a second-order ten-factor model, respectively. The lavaan R package was used to conduct CFA analyses. The construct validity was investigated using Principal Axis Factoring with Kaiser normalization.

### Ethical considerations

The study protocol was approved by the Princess Nourah University Institutional Review Board (IRP); the IRP number was (19–0105). Participants' consent in the focus group was acquired verbally and in writing. The research data were kept secure, and only the research team could access the focus group recording.

## Results

The study results were divided into two sections based on the pre-defined phases of the development and validation of the instrument. Each phase was dvided into two steps.

### Phase I: qualitative phase

#### Step 1: Instrument theoretical background

The purpose of the instrument was to assess the quality of healthcare simulation scenario components. An extensive literature review was done on all literature that discussed the quality of healthcare simulation scenarios to establish a framework for the instrument. Additionally, published or available templates were included in the literature review. The literature review findings indicated that six major domains determine the quality of the scenario. Those domains were: Learning objectives, patient's case, scenario setting, scenario flow, critical actions, and debriefing. Each domain was divided into sub-domains. The first domain, learning objectives, described that when writing the learning objectives, they must be formatted in SMART format (Specific, Measurable, Achievable, Relevant, and Time-Bound) [28–30]. A critical part of writing learning objectives was utilizing Bloom’s taxonomy [31, 32]. Additionally, learning objectives must be aligned with the learner's level [1, 14]. The second domain was the patient case, which focused on the patient's medical history, diagnosis, and demographic data [1, 33, 34].

The third domain was scenario setting, which included fidelity defined by the environment of the simulation event that matches the actual clinical setting and how the equipment and simulation modality utilized for the scenario imitate the clinical setting [1, 13, 28]. The fourth domain, scenario flow, focused on patient parameters progression appropriate to the learner's actions and prompting, ensuring a smooth transition of the scenario flow. The fifth domain (critical actions) was defined by the learner's actions required to achieve the scenario objectives and simulation outcomes [1, 12, 14–17]. The final domain was debriefing, and it was stated in the literature that debriefing time and method should be appropriate for the progression and level of the complexity scenario [13, 20]. Additionally, much literature focused on the facilitator experience and its effect on the scenario outcome and learning experience [13, 20].

Virtual focus groups were conducted with experienced simulation educators to discuss their opinions on the quality indicators of healthcare simulation scenarios and the domains found in the literature review. Three focus groups were conducted with 17 simulation educators with experience ranging from 2 to 15 years in simulation-based education. Participants involved exclusively in the operation of simulation activities only were excluded from the study [35]. The focus group questions were written based on an in-depth literature review of available articles describing the quality indicators of simulation scenarios in healthcare education [2, 6, 13]. The aim was to discuss quality indicators of simulation scenarios; questions asked revolved around the participants' experience in conducting simulation scenarios and their opinions of the factors considered important to scenario design. Mujlli et al. detail the qualitative study protocol and steps [34].

Participants were selected from the LinkedIn website based on the information provided on their public page and by recommendations from local simulation experts [36]. Constant comparison analysis was used to analyze the focus group audio recordings [27]. The analysis was done by two researchers and was reviewed by the research team during and after completion to detect inconsistency in findings [37].

**Fig. 2:**
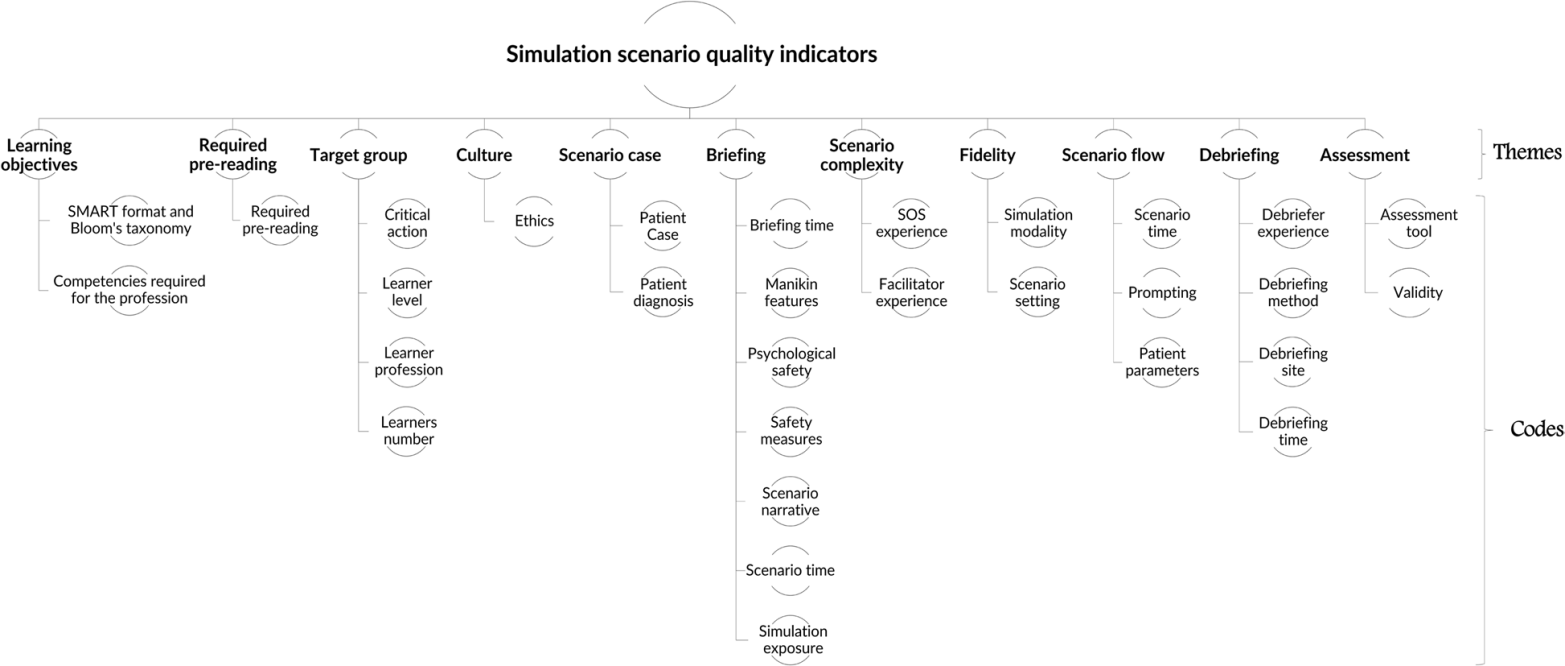
Constant comparison analysis results from the focus groups

The following themes were found after analyzing the focus group transcripts: learning objectives, required pre-reading, target group, culture, scenario case, briefing, scenario complexity, fidelity, scenario flow, debriefing, and assessment. Figure 2 shows the results of the constant comparison analysis of the focus groups.

#### Step 2: instrument construction

In the second step, the measurement instrument framework was written based on the established first step. The item writing was done based on both the established framework and the scale of the instrument and was chosen based on achieving the quality domain (Meets expectation (2), Needs improvement (1), Inadequate (0). The instrument underwent four rounds of review by the research team before finalizing the first version. The instrument had 55 items and 12 sections (Additional file 1: Appendix A).

After finalizing the first version of the instrument, it was sent to five experts in healthcare simulation for face and content validity. A copy of the instrument was sent to the experts, and a virtual interview was scheduled to discuss the expert's feedback. The interview thoroughly discussed the reviewer's feedback on each item and section in the instrument. Each expert was asked about their judgment regarding the relevancy of the item based on the following score: highly relevant (4), relevant (3), somewhat relevant (2), and not relevant (1). After summarizing the reviewers’ comments (Additional file 2: Appendix B), the S-CVI/Ave of the instrument was 0.87 (Additional file 3: Appendix C). The final step was revising the instrument and developing new items based on the expert's validation report. The score scale for the instrument was: Meets expectation (2), Needs improvement (1), Inadequate (0)).

### Phase (II): quantitative phase

#### Step 1: instrument piloting

The instrument was piloted among simulation educators in SSDC and one educator from outside the organization. The educators were assigned a specific number of scenarios to be reviewed and were free to choose from the scenarios in the SSDC scenario library or from scenarios they implemented in their simulation activities. The number of educators included in the piloting was seven from different specialties. Their experience in simulation ranged from 2 to 15 years in simulation design and conduction of simulation activities. Table 1 shows the piloting report of the SSQI.

**Table 1:** Piloting report of the simulation scenario quality instrument (SSQI)

			***p*-value**
**Scenario targeted profession** (*n* = 125) n (%)	**Allied health**	7 (5.6%)	** < 0.001**
	**Dentistry**	6 (4.8%)	
	**Medicine**	39 (31.2%)	
	**Nursing**	41 (32.8%)	
	**Pharmacy**	31 (24.8%)	
	**Interprofessional education (IPE)**	1 (0.8%)	
**Specialty of reviewer** (*n* = 7) n (%)	**Allied health**	2 (33.3%)	** < 0.001**
	**Medicine**	1 (1.1%)	
	**Nursing**	3 (50%)	
	**Pharmacy**	1 (16.7%)	
**Scenario score in the SSQI instrument** (*n* = 125) Mean ± Standard deviation (SD)	**Allied health (*n* = 7)**	64.86 ± 20.83	**0.017**
	**Dentistry (*n* = 6)**	87.33 ± 18.61	
	**Medicine (*n* = 39)**	85.54 ± 19.19	
	**Nursing (*n* = 41)**	73.66 ± 16.65	
	**Pharmacy (*n* = 39)**	75.55 ± 19.17	
	**Interprofessional education (IPE) (*n* = 1)**	81 ± NA^*^	

^*^NA Not available due to small sample size

#### Step 2: Instrument validation and reliability test

##### Construct validity and reliability analysis

The construct validity was investigated using Principal Axis Factoring with Kaiser normalization. For this analysis, the first step involved running a factor analysis on the items to ascertain the covariation among the items and whether the patterns fit well into the SSQI constructs. Based on the exploratory factor analysis (EFA) results, nine items with a factor loading of less than 0.3 were excluded from the instrument [38]. The factor analysis yielded eleven factors that explain the variance, which was less than the original framework of the instrument.

The internal reliability of the instrument was investigated using Cronbach's alpha [39]. Results indicated that the alpha for the total scale was 0.92. Examination of individual item statistics did not show the need to eliminate items to increase the scale's reliability (Additional file 4: Appendix D). After reviewing and editing the instrument, the factors were revised and renamed based on the general context of the items and the research team's input. Then, confirmatory factor analyses were conducted to investigate the construct validity of the revised instrument.

##### Confirmatory Factor Analyses (CFA) results

In this section, the construct validity of SSQI was examined by different confirmatory factor analysis (CFA) models. For this purpose, the one-factor CFA model, where all items in the survey load on one latent factor; the ten-factor CFA model, where each survey domain was treated as a factor; and the second-order CFA model were tested. Different fit measures were reported and used to assess model-data fit and to determine the best CFA model that fits the data.

The most commonly used fit measures are chi*-square statistics, CFI (the comparative fit index)*, TLI (the *Tucker-Lewis index*), and *RMSEA (root mean square error of approximation),* which provide insight into the degree of data fit for a given model. Different criteria for fit measures were proposed to evaluate the degree of model fits. Hu and Bentler (1999) proposed that an RMSEA less than 0.06 and CFA and TLI fit measures greater than 0.95 (RMSEA 0.06, CFA ≥ 0.95, and TLI ≥ 0.95) indicate a good fit [40]. Additionally, a less stringent criteria were proposed by Marsh, Hau, and Wen (2004) in which CFA ≥ 0.90, TLI ≥ 0.90, and RMSEA ≤ 0.08 indicate an acceptable model-data fit [41].

The CFA analyses were first conducted based on the initial factorial structure of SSQI that contained 40 items distributed across ten domains (Table 2). According to the one-factorial and ten-factorial CFA results, two items showed a poor fit to the model with factor loadings less than 0.30. Thus, these items with poor fit were excluded from the instrument, and then CFA models were tested again. Table 2 provides the results of CFA models.

**Table 2:** The results of one-factor, ten-factor, and second-order CFA models

Group	X^2^	df	CFI	TLI	RMSEA	90% for RMSEA	
						LL	UL
One-factor model	2151.856	665	0.985	0.985	0.134	0.128	0.141
**Ten-factor model**	**829.484**	**620**	**0.998**	**0.998**	**0.052**	**0.042**	**0.061**
Second-order model	1133.760	655	0.995	0.995	0.077	0.069	0.084

*Abbreviations*: *X*^*2*^ Chi-square, *df *Degree of freedom, *CFI* Comparative fit index, *TLI* Tucker-Lewis index, *RMSEA* Root mean square error of approximation

**Fig. 3:**
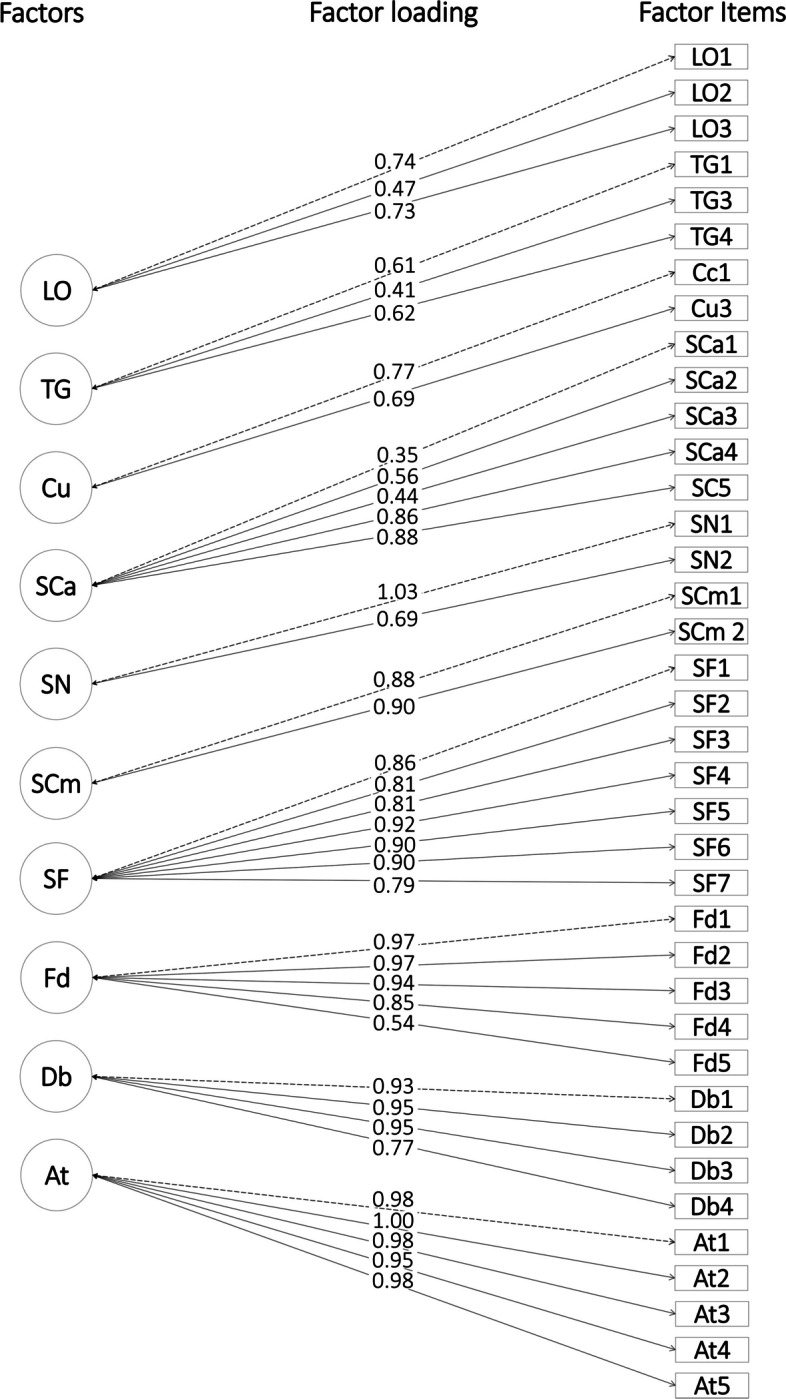
Path diagram of ten-factorial CFA model. Abbreviation: LO = Learning objectives; TG = Target group; Cu = Culture; SCa = Scenario case; SN = Scenario narrative briefing; SCm = Scenario complexity; SF = Scenario flow; Fd = Fidelity; Db = debriefing; At = assessment

The fit measures in Table 2 showed that CFI and TLI statistics were above 0.95 for all CFA models. However, RMSE values for one-factor and second-order models were higher than 0.06 except for the ten-factor model (RMSE = 0.052), indicating that it showed a better fit compared to the other two models. Additionally, investigating the factor loadings for the ten-factor model revealed that all items had factor loadings greater than 0.30 (Table 3). Moreover, since the ten-factor model had CFI and TLI fit measures greater than 0.95 and RMSE values less than 0.06, a good fit between the ten-factor CFA model and data was achieved, as Hu and Bentler (1999) suggested. These results indicate that the ten-factor CFA model with 38 items achieved a robust construct validity. The factor loadings and path diagram of the ten-factor model are provided in Table 3 and Fig. 3, respectively. Additionally, Table 4 provides the final version of the SSQI instrument after CFA analyses.

**Table 3:** Factor loadings of ten-factor CFA models

					**Factors**					
**Factor items**	**LO**	**TG**	**Cu**	**SCa**	**SN**	**SCm**	**SF**	**Fd**	**Dbr**	**AT**
LO1	0.739									
LO2	0.466									
LO3	0.726									
TG1		0.611								
TG3		0.411								
TG4		0.619								
Cu1			0.765							
Cu3			0.687							
SCa1				0.351						
SCa2				0.557						
SCa3				0.436						
SCa4				0.856						
SCa5				0.876						
SN1					1.033					
SN2					0.691					
SCm1						0.903				
SCm2						0.877				
SF1							0.861			
SF2							0.807			
SF3							0.814			
SF4							0.918			
SF5							0.898			
SF6							0.900			
SF7							0.791			
Fd1								0.969		
Fd2								0.970		
Fd3								0.942		
Fd4								0.854		
Fd5								0.542		
Dbr1									0.930	
Dbr2									0.980	
Dbr3									0.948	
Dbr4									0.772	
AT1										0.985
AT2										0.998
AT3										0.985
AT4										0.950
AT5										0.982

*Abbreviation*: *LO* Learning objectives, *TG* Target group, *Cu* Culture, *SCa* Scenario case, *SN* Scenario narrative briefing, *SCm* Scenario complexity, *SF* Scenario flow, *Fd* Fidelity, *Db* Debriefing, *At* Assessment

**Table 4:** Final version of Simulation Scenarios Quality Instrument (SSQI)

Scenario Element	Item	Meets Expectations (2)	Needs Improvement (1)	Inadequate (0)
**1. Learning objectives**	**1.1** Learning objectives are written according to SMART format^*^ ^*^ SMART: Specific, **M**easurable, **A**ttainable, **R**elevant, and **T**ime-bound	2	1	0
	**1.2** Learning objectives are written according to Bloom’s taxonomy^*^ ^*^ Bloom’s taxonomy is a framework consisting of six major categories: knowledge, comprehension, application, analysis, synthesis, and evaluation. Each category has its action verbs that are used in writing learning objectives	2	1	0
	**1.3** Required pre-reading materials provided in the scenario are related to the learning objectives	2	1	0
**2. Target group**	**2.1** Learner prerequisite knowledge and skills is stated clearly in the scenario	2	1	0
	**2.2** Learners’ number is appropriate for the scenario conduction (the instructor-to-learner ratio is based on best practice)	2	1	0
	**2.3 (**Critical actions are part of the competencies required for the learner’s profession	2	1	0
**3. Culture**	**3.1** The scenario is compatible with local laws and regulation of the healthcare system	2	1	0
	**3.3** Patient resembles common demographic information to the local population	2	1	0
**4. Scenario case**	**4.1 The demographic information (such as age, gender, and religion) are stated clearly in the scenario case**	2	1	0
	**4.2 The anthropometric measurements (such as weight, height, and BMI) are stated clearly in the scenario case**	2	1	0
	**4.3** Medical history is stated clearly in the scenario case	2	1	0
	**4.4** Patient current health status is stated clearly in the scenario case	2	1	0
	**4.5** Initial physical examination findings are stated clearly in the scenario case	2	1	0
**5. Scenario narrative briefing**	**5.1 The briefing elements (such as psychological safety, confidentiality agreement…) have been addressed in the briefing section:**	2	1	0
	**5.2** Briefing time stated is enough to brief the students about the briefing elements	2	1	0
**6. Scenario complexity**	**6.1** The distractors provided in the scenario flow do not negatively impact achieving learning objectives	2	1	0
	**6.2** The complexity of the scenario matches learner level	2	1	0
**7. Scenario flow**	**7.1** Patient parameters and/or status are aligned with the initial statues stated in the scenario case	2	1	0
	**7.2** Patient parameters and/or status progresses according to leaner\s actions	2	1	0
	**7.3** Scenario flow indicate appropriate prompting for leaners who do not progress according to the indicated time	2	1	0
	**7.4** The simulation flow and overall scenario outline are clear	2	1	0
	**7.5** The progression of scenario flow is realistic	2	1	0
	**7.6** Scenario flow time is adhering to center’s guidelines (if no guidelines available, scenario should not exceed 25 min)	2	1	0
	**7.7** Stated learner’s actions include critical actions stated in the “Critical action” section	2	1	0
**8. Fidelity**	**8.1** The physical context of simulation-based activity replicates the actual environment (e.g., simulator, equipment, environment, Moulage etc.) ***(Physical fidelity)***	2	1	0
	**8.2** Required equipment and simulators were detailed	2	1	0
	**8.3** Elements of the scenario are related to the scenario flow (e.g., vital signed are similar to the patient diagnosis) ***(conceptual fidelity)***	2	1	0
	**8.4** The script provided for the SP and direction of training is clearly stated (If applicable)	2	1	0
	**8.5** The moulage picture is related to the scenario case (If applicable)	2	1	0
**9. Debriefing**	**9.1** Appropriate debriefing method is identified to cover the objectives of the simulation session	2	1	0
	**9.2** Debriefer experience stated and compatible with the skills level required to implement the debriefing method	2	1	0
	**9.3** Debriefing site is stated and is appropriate for the scenario	2	1	0
	**9.4** Debriefing time is sufficient to conduct a comprehensive session	2	1	0
**10. Assessment**	**10.1** Assessment tool cover all of the scenario’s learning objectives	2	1	0
	**10.2** The assessment tool items are measurable and observable	2	1	0
	**10.3** All targeted critical actions, and/or skills, procedures are addressed in the assessment tool	2	1	0
	**10.4** The assessment tool grading system is clear	2	1	0
	**10.5** Assessment tool used is validated (Optional)	2	1	0

## Discussion

This study described the process of developing and validating the SSQI for evaluating the quality of healthcare simulation scenarios [25]. Only one published tool was developed to evaluate the simulation scenarios (SSET) [22]. To the authors’ knowledge, this is the second attempt to develop and validate an instrument for assessing the quality of healthcare scenarios. The internal reliability of the instrument was measured using Cronbach’s alpha. The results indicated that the alpha for the total scale was 0.92 [39]. The results of the content validation showed a moderate agreement with the components of the healthcare simulation scenario that the instrument should assess: Scenario case, culture, patient demographic information, patient medical information, environment fidelity, patient fidelity, and debriefing. The final version of the instrument included factors and items consistent with several guidelines and research that investigated the elements of simulation scenarios that included the final elements of the instrument [2, 10, 42].

According to Lioce et al. best practice of simulation design, the SSQI elements 8 of the 11 elements listed as a framework for developing effective simulation scenarios [10]. The elements included measurable objectives, simulation format, clinical scenario or case, fidelity, facilitative and facilitator approach, briefing, debriefing, and evaluation [10]. Another study investigated the quality indicators for developing and implementing simulation experiences using the Delphi method [28]. Two of the quality indicators were aligned with the final elements of SSQI, which included all elements listed in the study findings. The “Pedagogical principles” indicator stated that simulation experiences should align with the curriculum, alignment between the program and the simulation, and learning objectives stated in elements one and two: learning objectives target group. The second indicator, “Fidelity,” noted that the simulation technology and environmental fidelity should be aligned with the learning objectives stated in the items under the same name [28].

There are recent studies that described a similar framework to developing simuation epxirnces. In Hewart et al. study, the process of designing simulation-based experiences for speech-language pathology was listed, and it included the development of simulation scenarios based on Lioce et al. 2015 work, which was referenced above, and the framework was recommended for other disciplines [43]. Another recent study described the steps required to develop simulation scenarios, emphasizing the most relevant aspect of the design [44]. The steps listed were all found in the SSQI tool: objectives, simulation format, case description, realism, pre-debriefing, debriefing, and evaluation [44].

Multiple simulation scenario templates were developed to assist educators with developing evidence-based simulation scenarios. The SSQI has similar elements to the developed templates. In Munroe et al. study, the authors devised a new simulation scenario template for research purposes [45]. Elements included in the new template were similar to the SSQI quality indicators, which included: Modality and room setup (which was defined in SSQI as fidelity), Patient profile (sncario case in SSQI), narrative description of the scenario, physiological parameters and patient progress (which was scenario flow in SSQI), and post-simulation debriefing [45]. Another template was developed in 2015 for critical simulation called the Template of Events for Applied and Critical Healthcare Simulation (TEACH Sim) [14]. This template aimed to assist educators and clinicians in developing simulation scenarios and overcoming the potential challenges they might face. The template sections were designed in a way that is similar to the SSQI; however, it used different phrasing. The learning objectives in SSQI were the same as in TEACH Sim. However, the scenario case in SSQI was written in a Clinical context. Scenario case was also patient profile, while fidelity was divided into modality and equipment props [14].

A similar tool was developed in 2019 to evaluate the quality of simulation scenarios. The “Simulation Scenario Evaluation Tool (SSET)” was developed in 2019 using the modified Delphi method. The instrument was developed by reviewing the literature and based on published simulation scenario design templates and developing the instrument to include six components of scenario quality with corresponding scores and anchors. Then, the tool was sent to a national group of experts to demonstrate a consensus on the final assessment instrument. The instrument was validated by simulation educators using content validity and showed a significant level of agreement (*p* < 0.05). The instrument went through a two-round Delphi approach; the first round included 38 complete responses, and the second round included 22 complete responses. The SSQI instrument was developed using a different method, and content and construct validity were tested. Content validity was defined using the average content validity index, which was 0.87. SSQI was also tested for construct validity and showed a good fit to the proposed model developed after researching simulation design best practices and content experts in simulation from different experience levels and clinical backgrounds. The Cronbach alpha of the instrument was 0.92.

Scenario design is a complex process, and it is recommended that simulation experts use published templates to assist in writing healthcare simulation scenarios [1]. The majority of the feedback and reviews of the scenarios are objective and not structured [22]. The only instrument found in the literature that evaluates written simulation scenarios was the SEET instrument, which, while it is the first instrument to assess simulation scenario quality, the authors noted that it is an instrument validated by content experts and the current instrument use multiple arguments of validity, content validity, and construct validity [22]. The need for validated instruments supports the importance of developing a validated assessment instrument to determine the quality of healthcare simulation scenarios.

## Limitations

The study has some limitations that need to be addressed. First, this instrument was not piloted again after conducting the construct validity in phase IV. The second limitation was the limited evaluation of simulation scenarios included in the pilot and the limited number of reviewers who utilized the instrument in the pilot. Finally, no cut-off points were established to determine the levels of quality that each final score indicates.

## Conclusions

The validity and reliability analysis results imply that the SSQI is a valid and reliable instrument developed to assess the quality of healthcare simulation scenarios. This tool provides the simulation educators and scenario writers with the expected elements detrimental to designing high-quality scenarios. It is recommended for future research to conduct a second pilot of this instrument and includes a larger pool of subjects to investigate inter-rater reliability among raters.

### Supplementary Information

**Additional file 1:** **Appendix A.** Simulation Scenarios Quality Instrument (SSQI) – Version 1.

**Additional file 2:** **Appendix B.** Content validity report of the simulation scenario quality instrument (SSQI).

**Additional file 3:** **Appendix C.** The relevance ratings on the item scale by five experts.

**Additional file 4:** **Appendix D.** Factor matrix of SSQI items and Cronbach alpha score if the item was deleted.

## Data Availability

The databases used and analyzed during the study are available from the corresponding author upon reasonable request.

## Abbreviations

At: Assessment
CFA: Confirmatory factor analysis
CFI: Comparative fit index
Cu: Culture
Db: Debriefing
df: Degree of freedom
EFA: Exploratory factor analysis
Fd: Fidelity
I-CVI: Item content validity index
IRP: Institutional Review Board
LO: Learning objectives
RMSEA: Root mean square error of approximation
SBL: Simulated-Based Learning
SBT: Simulation-based training
SCa: Scenario case
SCm: Scenario complexity
S-CVI: Item content validity index scores
SF: Scenario flow
SMART: Specific, Measurable, Achievable, Relevant, Time-Bound
SN: Scenario narrative briefing
SSDC: Simulation and Skills Development Center
SSET: Simulation Scenario Evaluation Tool
SSQI: Simulation Scenario Quality Instrument
TG: Target group
TLI: Tucker-Lewis index
X2: Chi-square
