# Supplementary material for: Development and validation of Simulation Scenario Quality Instrument (SSQI)
Source: BMC Med Educ. 2023 Dec 19;23:972. doi: 10.1186/s12909-023-04935-5 (PMC10731859; doi:10.1186/s12909-023-04935-5)
Supplement: Supplementary file 1 — Additional file 1: Appendix A. Simulation Scenarios Quality Instrument (SSQI) – Version 1. [file 12909_2023_4935_MOESM1_ESM.docx]

**Introduction:** This instrument aims to evaluate the quality of healthcare simulation scenarios. Rate the scenario elements according to the scale showed in the table below:

| **Scenario Element** | **Item** | **Meets Expectations (2)** | **Needs Improvement (1)** | **Inadequate (0)** |
| --- | --- | --- | --- | --- |
| 1. **Learning objectives** | **1.1** Learning objectives are written according to SMART format |  |  |  |
|  | **1.2** Learning objectives are written according to Bloom’s taxonomy. |  |  |  |
|  | **1.3** Learning objectives are part of the competencies required for this profession |  |  |  |
| 1. **Required pre-reading** | **2.1** Required pre-reading materials provided in the scenario are related to the learning objectives |  |  |  |
| 1. **Target group** | **3.1** Learner prerequisite knowledge and skills (level differs among curriculums and countries is stated clearly in the scenario |  |  |  |
|  | **3.2** Learner profession is stated clearly in the scenario |  |  |  |
|  | **3.3** Learners number is appropriate for the scenario conduction (instructor to learner ratio is based on best practice) |  |  |  |
| 1. **Critical action** | **4.1** Critical actions are part of the competencies required for the learner’s profession. |  |  |  |
| 1. **Culture** | **5.1** The scenario is compatible with local laws and regulation of the healthcare system. |  |  |  |
|  | **5.2** the scenario follows the center’s or site code of conduct and ethical standards. |  |  |  |
|  | ***Note: Does the scenario aims to tackle cultural topics, domains, themes?***  *Yes (If “Yes” Fill litem 5.3)  No (If “No” Skip item 5.3)*  **5.3** The patient resembles common demographic information to the local population? |  |  |  |
| 1. **Scenario case** | **6.1 The following demographic information are stated clearly in the scenario case (If applicable):**  **6.1.1** Name |  |  |  |
|  | **6.1.2** Age |  |  |  |
|  | **6.1.3** Gender |  |  |  |
|  | **6.1.4** Marital statues (If applicable) |  |  |  |
|  | **6.1.5** Religion (If applicable) |  |  |  |
|  | **6.1.6** Ethnicity (If applicable) |  |  |  |
|  | **6.1.7** Occupation (If applicable) |  |  |  |
|  | **6.2 The following anthropometric measurement are stated clearly in the scenario case (If needed):**  **6.2.1** Weight |  |  |  |
|  | **6.2.2** Height |  |  |  |
|  | **6.2.3** Body mass index (BMI) |  |  |  |
|  | **6.3** Medical history is stated clearly in the scenario case |  |  |  |
|  | **6.4** Patient current status is stated clearly in the scenario case |  |  |  |
|  | **6.5** Physical examination findings are stated clearly in the scenario case |  |  |  |
| 1. **Scenario narrative** | **7.1 The following elements in scenario narrative are stated clearly in the scenario case:**  **7.1.1** Location of the case |  |  |  |
|  | **7.1.2** Time of the case |  |  |  |
|  | **7.1.3** Patient current status |  |  |  |
|  | **7.1.4** Case background (SBAR) |  |  |  |
| 1. **Briefing** | **8.1 The following briefing elements have been addressed in the briefing section:**  **8.1.1** Psychological safety |  |  |  |
|  | **8.1.2** Safety measures |  |  |  |
|  | **8.1.3** Confidentiality agreement |  |  |  |
|  | **8.1.4** Scenario narrative |  |  |  |
|  | **8.1.5** Scenario time |  |  |  |
|  | **8.2** Briefing time stated is enough to brief the students about the briefing elements. |  |  |  |
| 1. **Scenario complexity** | **9.1** The distractors provided in the scenario flow do not negatively impact achieving objectives. |  |  |  |
|  | **9.2** The complexity of the scenario matches leaner level. |  |  |  |
| 1. **Scenario flow** | **10.1** Patient parameters and/or status are aligned with the initial statues stated in the scenario case. |  |  |  |
|  | **10.2** Patient parameters and/or status progresses according to leaner\s actions. |  |  |  |
|  | **10.3** Scenario flow indicate appropriate prompting for leaners who do not progress according to the indicated time. |  |  |  |
|  | **10.4** The simulation flow and overall scenario outline is clear and comprehensive. |  |  |  |
|  | **10.5** The progression of scenario flow is realistic and transition between steps is seamless. |  |  |  |
|  | **10.6** Scenario flow time is adhering to center’s guidelines (if no guidelines available, scenario should not exceed 25 minutes). |  |  |  |
|  | **10.7** Stated learner’s actions include critical actions stated in the “Critical action” section. |  |  |  |
| 1. **Fidelity** | **11.1** The physical context of simulation-based activity replicates the actual environment (e.g. simulator, equipment, environment etc.) ***(Physical fidelity)*** |  |  |  |
|  | **11.2** Elements of the scenario case are related to the scenario flow (e.g. vital signed are similar to the patient diagnosis) ***(conceptual fidelity)*** |  |  |  |
| 1. **Debriefing** | **12.1** Well known debriefing method is identified to cover the objectives of the simulation session. |  |  |  |
|  | **12.2** Debriefer experience stated and compatible with the skills level required to implement the debriefing method. |  |  |  |
|  | **12.3** Debriefing site is stated and is appropriate for the scenario. |  |  |  |
|  | **12.4** Debriefing time is sufficient to conduct a comprehensive session. |  |  |  |
| 1. **Assessment** | **13.1** Assessment tool cover all of the scenario’s learning objectives. |  |  |  |
|  | **13.2** The assessment tool items are measurable and observable. |  |  |  |
|  | **13.3** All targeted critical actions, and/or skills, procedures are addressed in the assessment tool. |  |  |  |
|  | **13.4** The scale/ marking/ grading system is clear and feasible. |  |  |  |
|  | **13.5** Assessor are oriented to the assessment tool. |  |  |  |
|  | **13.6** Assessment tool used is validated (Optional). |  |  |  |
| **Total score** | |  | | |
