# Supplementary material for: Development and validation of Simulation Scenario Quality Instrument (SSQI)
Source: BMC Med Educ. 2023 Dec 19;23:972. doi: 10.1186/s12909-023-04935-5 (PMC10731859; doi:10.1186/s12909-023-04935-5)
Supplement: Supplementary file 2 — Additional file 2: Appendix B. Content validity report of the simulation scenario quality instrument (SSQI). [file 12909_2023_4935_MOESM2_ESM.docx]

**Appendix B: Content validity report of the simulation scenario quality instrument (SSQI)**

| **Instrument sections and items** | **Experts (1)** | **Experts (2)** | **Experts (3)** | **Experts (4)** |
| --- | --- | --- | --- | --- |
| Overall decision | **General comments:** The tool seems directed to specific types of cases, and contains some items that cannon be verified from the scenario sheet (needs implementation to judge). I think simulation experts from multiple fields (family medicine for example) need to be involved in the item creation. Also, this does not cover SPs and their training / instructions  **Additional suggestion:** Take some items from this checklist and make another checklist for scenario implementation.  Make the tool general to target all scenarios, or narrow the scope for different target groups. | **General comments:** The tool in innovative, and it’s covered all details related to simulation scenarios. Name of the instrument needs to be catchier.  Additional suggestions: I suggest that the tool is divided into two parts, the first part can be reviewed by the subject matter expert and the second part is covered by simulation expert. This way it’s easier to track than adding the section numbers at the beginning of the instrument. | **General comments:** There is no mention of faculty required to run the scenario, I would suggest to add that. Ideally an operation person, a facilitator, an valuator/de-briefer and confederate if required INACSL Simulation facilitation (criterion 1).  Add faculty required, operational team, staff required, in the template and instrument.  **Additional suggestion:** Add a sentence that states that this instrument covers all simulation events, activities.  Defining (Scenario) might not cover all the elements stated below, find a different word that better describe the simulation event. | **General comments:**  Clear introduction. |
| Scale | Inadequate and needs improvement overlap, maybe "missing"  - maybe add N/A (with full score) | Scoring for domains with multiple items needs to be specified: it can be written in each box where the scenario evaluator can simply circle the numbers and calculate | No comment | No comment on the grading system, not applicable should be added as a separate column. |
| Item (1.1) | Redundant, the reason we use bloom's taxonomy is to make it measurable  Combine 1.1 with 1.2 | Good,  It should be added to the simulation educator section | Nicely aligned with INACSL Simulation outcome and objectives 2 | No comment |
| Item (1.2) | No comment | I would suggest a line description of bloom’s taxonomy as a reminder for the evaluator who will be using this instrument to assess sim scenario quality.  It should be added to the simulation educator section | Nicely aligned with INACSL Simulation outcome and objectives 2 | No comment |
| Item (1.3) | (If applicable?)  What if the scenario does not require it? | Competencies could be replaced by learning outcomes (Comment changed on second review)  Clear | Nicely aligned with INACSL Simulation outcome and objectives 2 | “The Simulation scenario covers the desired objectives for the course”  Instead of this item write the above statement.  Delete this item as it not needed because it’s expected to have the learning objectives customized for the target audience. |
| Item (2.1) | Maybe link 3.1 to 2.1 (if no then material is required) | Good | I am not sure what is the word pre reading means here?  If it means learning materials for learner or tutor prior to simulation than rephrase as Required pre-simulation/preparatory learning materials provided/sign posted in the scenario and are aligned with learning objectives  INACSL Simulation design criterion 7  INACSL Simulation facilitation criterion 3  Reviewer recommended  (Sign posted)  Pre-reading might convey a flipped classroom approach rather than simulation scenario | No comment  Leave as is |
| Item (3.1) | No comment | Not sure if the highlighted part is needed as it is obvious (redundant) | Learner’s prerequisite knowledge and skills level clearly stated (differs among curriculums and countries is stated clearly in the scenario)  By default, it’s understood (Universal truth) | How?  Delete highlighted section  (level differs among curriculums and countries is stated clearly in the scenario) |
| Item (3.2) | No comment | Good | Learner’s profession | No comment |
| Item (3.3) | What if there aren’t critical actions? (Maybe add if applicable)  Sometime there are no clear critical action (critical dilemma) | Important | Learner’s number | No comment  AHL is the reference (for life support courses) |
| Item (4.1) | Move this section to after section 6 (Scenario Case) | Important | Agree, no comments | No comment |
| Item (5.1) | No comment | Good | Agree, no comments | No comment |
| Item (5.2) | No comment | Good | Agree, no comments | No comment |
| Item (5.3) | No comment | Clear | Agree, no comments | No comment |
| Item (6.1.1) | No comment | Good  This section should be divided into two section , one is the mandatory items (6.1.1-6.1.3) and section for the optional items (6.1.4-6.1.7) and the heading is “Documented the required of the following (if applicable) “ and it will be marked as not applicable if it’s not filled | Nicely aligned with INACSL Simulation design criterion 7 | No comment |
| Item (6.1.2) | No comment | Good | As above | No comment |
| Item (6.1.3) | No comment | Good | As above | No comment |
| Item (6.1.4) | No comment | Good | As above | No comment |
| Item (6.1.5) | No comment | Ok | As above | This might be mandatory e.g. maternity scenario to how to deal with newborn and mother |
| Item (6.1.6) | No comment | Ok | As above | No comment |
| Item (6.1.7) | No comment | Important | As above | No comment |
| Item (6.2.1) | No comment | Important | As above | Mandatory |
| Item (6.2.2) | No comment | Ok | As above | Mandatory |
| Item (6.2.3) | Why BMI?, Should be not applicable or deleted | Ok, add (If applicable) | As above | Mandatory (to reduce calculation time) |
| Item (6.3) | No comment | Important | As above | No comment |
| Item (6.4) | No comment | Important | As above | No comment |
| Item (6.5) | Initial | Important | As above | No comment |
| Item (7.1.1) | No comment | Better to replace the word case by encounter | As above | Important |
| Item (7.1.2) | No comment | Time of encounter | As above | Important |
| Item (7.1.3) | Redundant (repeated) | Important | As above (SBAR) add in both 7.1.3 and 7.1.4 | Important |
| Item (7.1.4) |  | Important | As above | Important |
| Item (8.1.1) | No comment | Important  Items 8.1.1 – 8.1.3 should only mentioned I the briefing section without details | Nicely aligned with INACSL Simulation design criterion 7  This includes both pre-briefing and briefing | Important  Should be written in details |
| Item (8.1.2) | No comment | Important | As above | Important  Should be written in details |
| Item (8.1.3) | Time consuming to write all those details in the scenario template.  8.1-2-3, can be mentioned without specifics on the scenario document/template | Important | As above | Important |
| Item (8.1.4) | No comment | Important | As above | Important |
| Item (8.1.5) | No comment | Important | Also add a point about “orientation of participants to the simulation environment (manikins, equipment’s etc.)  INACSL Simulation facilitation criterion 3 | Important  When and How scenario will be initiated () |
| Item (8.2) | No comment | Important | Agree, no comments | No comment |
| Item (9.1) | Are appropriate for target learner level | Important, Clear  Good  Add “learning” before “Objectives” | Agree, no comments | No comment |
| Item (9.2) | Which make this redundant  Nee more research on this section. | Important  This item can’t be standardized due to subjectivity  Not clear if the learning outcomes are not regulated | Agree, no comments | No comment |
| Item (10.1) | No comment | Important | Agree, no comments  Revise based in 1.1.2 comment | No comment |
| Item (10.2) | No comment | Good | Agree, no comments | No comment |
| Item (10.3) | No comment | Good | Nicely aligned with INACSL Simulation facilitation criterion 4 but I would suggest focusing on prompt here as flow has been address many times, rephrase as  “Predetermined cues (Instead of prompt) are incorporated in scenario design i.e. Data- (labs, Radiology), Verbal (phone call, confederate, patient or family member’s trigger) or visual (change in vital signs on monitor) etc.)”  Remove item and add the suggested sentence | No comment |
| Item (10.4) | Fragmented (evaluating 2 items in one score)  Either separate the two words (Clear, Comprehensive) or delete one word. | Good | Seems extra as flow and overall outline is already covered in narrative and 10.1&10.5  Remove | No comment |
| Item (10.5) | (Also 2 items in one)  Suggestion: I suggest adding a point (the scenario transitions cover most expected learner reactions) or Scenario time is appropriate to the learning objectives (Make it universal | Good | The progression of scenario flow and phases are realistic and transition between them is steps is seamless. | No comment  Add explanation about this item: Transition between phases (Time to move between steps).  How you end the scenario? |
| Item (10.6) | Different scenarios require different timings even in the same center | Important as we see many scenarios with no specific time and when applied it takes up to 2 hr!!!!  Keep as is  Time is important and regularly ignored when designing scenarios | Agree, no comments | No comment  15 minutes is enough for the scenario |
| Item (10.7) | No comment | Good | Agree, no comments | No comment |
| Item (11.1) | Can you show this in the scenario sheet? | Important | Nicely aligned with INACSL Simulation design criterion 5  Add moulage as well | I would prefer to add id SPs are involved we have to make sure they get proper training before the simulation course and scenario. Also, if moulage was applied, it has to be as realistic as possible.  Add item about SP, whenever involved they should have the proper training, the item could state that the script is provided for the SP and direction of training is clearly stated (Item is optional).  Add item about Moulage, the item could focus on the realism of the moulage and how related it is to the scenario case. (Item is optional) |
| Item (11.2) | No comment | Signs | 10.1 covers but ok as more obvious here  Revise |  |
| Item (12.1) | What defines well-known?  Suggestion: “Appropriate” instead of “Well-known” | Important | INACSL debriefing criterion 4 | No comment |
| Item (12.2) | No comment | Good | INACSL debriefing criterion 1 | No comment |
| Item (12.3) | No comment | Good (Optional in the template) | INACSL debriefing criterion 2 | No comment |
| Item (12.4) | No comment | Important | INACSL debriefing criterion 2 | No comment |
| Item (13.1) | No comment | Important  Assessment is not part of training \| (Not applicable), therefore it’s not mandatory to do when the scenario is designed for training purposes  Suggestion: add “If you are using an assessment tool, complete the following items” | It will be nice to mention to put one more point “method/ purpose of evaluation clearly identified (formative/ summative/ high stake)  INACSL Evaluation criterion 1  Suggestion on edits:  If the scenario is for training skip, if not, complete the following section | Mandatory section  Evaluation of the full simulation experience (360˚)  Important |
| Item (13.2) | Assessment is not always a part of simulation scenarios  2 items in one. Separate | Important | Agree, no comments | Important |
| Item (13.3) | Some overlap with 13.1.  Revise | Important | Agree, no comments | Important |
| Item (13.4) | Fragmented. Delete “and feasible” | Important | Agree, no comments | Important |
| Item (13.5) | Can you show that in the scenario sheet? | Good | How will the scenario tell that the assessor is oriented or not?  Remove | Important |
| Item (13.6) | I don't think it should be optional. (Required) | Good  Not optional, delete or revise to include newly developed instruments | Agree, no comments  Remove (It’s validated if it’s for summative assessment) | Important |
