# Supplementary material for: Development and validation of Simulation Scenario Quality Instrument (SSQI)
Source: BMC Med Educ. 2023 Dec 19;23:972. doi: 10.1186/s12909-023-04935-5 (PMC10731859; doi:10.1186/s12909-023-04935-5)
Supplement: Supplementary file 3 — Additional file 3: Appendix C. The relevance ratings on the item scale by five experts. [file 12909_2023_4935_MOESM3_ESM.docx]

**Appendix (C): The relevance ratings on the item scale by five experts**

| **Scenario Element** | **Item** | **Instrument code** | **Relevancy score ^*^** | | | | | **Item content validity index (I-CVI) ^**^** |
| --- | --- | --- | --- | --- | --- | --- | --- | --- |
|  |  |  | **Experts (1)** | **Experts (2)** | **Experts (3)** | **Experts (4)** | **Expert (5)** |  |
| **1. Learning objectives** | 1.1 Learning objectives are written according to SMART format | **Item (1.1)** | **1** | **3** | **4** | **4** | **4** | **0.80** |
|  | 1.2 Learning objectives are written according to Bloom’s taxonomy. | **Item (1.2)** | **4** | **4** | **4** | **4** | **4** | **1.00** |
|  | 1.3 Learning objectives are part of the competencies required for this profession | **Item (1.3)** | **3** | **4** | **4** | **1** | **4** | **0.80** |
| **2. Required pre-reading** | 2.1 Required pre-reading materials provided in the scenario are related to the learning objectives | **Item (2.1)** | **2** | **3** | **4** | **4** | **3** | **0.80** |
| **3. Target group** | 3.1 Learner prerequisite knowledge and skills (level differs among curriculums and countries is stated clearly in the scenario) | **Item (3.1)** | **3** | **3** | **3** | **3** | **4** | **1.00** |
|  | 3.2 Learner profession is stated clearly in the scenario | **Item (3.2)** | **4** | **4** | **4** | **4** | **4** | **1.00** |
|  | 3.3 Learners number is appropriate for the scenario conduction (instructor to learner ratio is based on best practice) | **Item (3.3)** | **4** | **4** | **4** | **4** | **2** | **0.80** |
| **4. Critical action** | 4.1 Critical actions are part of the competencies required for the learner’s profession. | **Item (4.1)** | **4** | **4** | **4** | **4** | **4** | **1.00** |
| **5. Culture** | 5.1 The scenario is compatible with local laws and regulation of the healthcare system. | **Item (5.1)** | **4** | **4** | **4** | **4** | **4** | **1.00** |
|  | 5.2 the scenario follows the center’s or site code of conduct and ethical standards. | **Item (5.2)** | **4** | **4** | **4** | **4** | **4** | **1.00** |
|  | Note: Does the scenario aims to tackle cultural topics, domains, themes?  ☐ Yes (If “Yes” Fill item 5.3)  ☐ No (If “No” Skip item 5.3) 5.3 The patient resembles common demographic information to the local population? | **Item (5.3)** | **4** | **4** | **4** | **4** | **4** | **1.00** |
| **6. Scenario case** | 6.1 The following demographic information are stated clearly in the scenario case (If applicable): 6.1.1 Name | **Item (6.1.1)** | **4** | **3** | **4** | **4** | **4** | **0.80** |
|  | 6.1.2 Age | **Item (6.1.2)** | **4** | **3** | **4** | **4** | **4** | **0.80** |
|  | 6.1.3 Gender | **Item (6.1.3)** | **4** | **3** | **4** | **4** | **4** | **0.80** |
|  | 6.1.4 Marital statues (If applicable) | **Item (6.1.4)** | **4** | **3** | **4** | **4** | **4** | **0.80** |
|  | 6.1.5 Religion (If applicable) | **Item (6.1.5)** | **4** | **3** | **4** | **4** | **4** | **0.80** |
|  | 6.1.6 Ethnicity (If applicable) | **Item (6.1.6)** | **4** | **3** | **4** | **4** | **4** | **0.80** |
|  | 6.1.7 Occupation (If applicable) | **Item (6.1.7)** | **4** | **4** | **4** | **4** | **4** | **1.00** |
|  | 6.2 The following anthropometric measurement are stated clearly in the scenario case (If needed): 6.2.1 Weight | **Item (6.2.1)** | **4** | **4** | **4** | **4** | **4** | **1.00** |
|  | 6.2.2 Height | **Item (6.2.2)** | **4** | **3** | **4** | **4** | **4** | **0.80** |
|  | 6.2.3 Body mass index (BMI) | **Item (6.2.3)** | **2** | **3** | **4** | **4** | **4** | **0.80** |
|  | 6.3 Medical history is stated clearly in the scenario case | **Item (6.3)** | **4** | **4** | **4** | **4** | **4** | **1.00** |
|  | 6.4 Patient current status is stated clearly in the scenario case | **Item (6.4)** | **4** | **4** | **4** | **4** | **4** | **1.00** |
|  | 6.5 Physical examination findings are stated clearly in the scenario case | **Item (6.5)** | **4** | **4** | **4** | **4** | **4** | **1.00** |
| **7. Scenario narrative** | 7.1 The following elements in scenario narrative are stated clearly in the scenario case: 7.1.1 Location of the case | **Item (7.1.1)** | **4** | **3** | **4** | **4** | **4** | **0.80** |
|  | 7.1.2 Time of the case | **Item (7.1.2)** | **4** | **3** | **4** | **4** | **4** | **0.80** |
|  | 7.1.3 Patient current status | **Item (7.1.3)** | **1** | **4** | **4** | **4** | **4** | **0.80** |
|  | 7.1.4 Case background (SBAR) | **Item (7.1.4)** | **3** | **4** | **4** | **4** | **4** | **0.80** |
| **8. Briefing** | 8.1 The following briefing elements have been addressed in the briefing section: 8.1.1 Psychological safety | **Item (8.1.1)** | **4** | **4** | **4** | **4** | **3** | **0.80** |
|  | 8.1.2 Safety measures | **Item (8.1.2)** | **4** | **4** | **4** | **4** | **3** | **0.80** |
|  | 8.1.3 Confidentiality agreement | **Item (8.1.3)** | **2** | **4** | **4** | **4** | **3** | **0.80** |
|  | 8.1.4 Scenario narrative | **Item (8.1.4)** | **4** | **4** | **4** | **4** | **3** | **0.80** |
|  | 8.1.5 Scenario time | **Item (8.1.5)** | **4** | **4** | **4** | **4** | **3** | **0.80** |
|  | 8.2 Briefing time stated is enough to brief the students about the briefing elements. | **Item (8.2)** | **4** | **4** | **4** | **4** | **3** | **0.80** |
| **9. Scenario complexity** | 9.1 The distractors provided in the scenario flow do not negatively impact achieving objectives. | **Item (9.1)** | **4** | **4** | **4** | **4** | **2** | **0.80** |
|  | 9.2 The complexity of the scenario matches leaner level. | **Item (9.2)** | **1** | **4** | **4** | **4** | **4** | **0.80** |
| **10. Scenario flow** | 10.1 Patient parameters and/or status are aligned with the initial statues stated in the scenario case. | **Item (10.1)** | **4** | **4** | **4** | **4** | **4** | **1.00** |
|  | 10.2 Patient parameters and/or status progresses according to leaner\s actions. | **Item (10.2)** | **4** | **3** | **4** | **4** | **4** | **0.80** |
|  | 10.3 Scenario flow indicate appropriate prompting for leaners who do not progress according to the indicated time. | **Item (10.3)** | **4** | **3** | **4** | **4** | **2** | **0.80** |
|  | 10.4 The simulation flow and overall scenario outline is clear and comprehensive. | **Item (10.4)** | **3** | **3** | **1** | **4** | **4** | **0.80** |
|  | 10.5 The progression of scenario flow is realistic and transition between steps is seamless. | **Item (10.5)** | **3** | **3** | **4** | **4** | **2** | **0.80** |
|  | 10.6 Scenario flow time is adhering to center’s guidelines (if no guidelines available, scenario should not exceed 25 minutes). | **Item (10.6)** | **2** | **4** | **4** | **4** | **4** | **0.80** |
|  | 10.7 Stated learner’s actions include critical actions stated in the “Critical action” section. | **Item (10.7)** | **4** | **4** | **4** | **4** | **4** | **1.00** |
| **11. Fidelity** | 11.1 The physical context of simulation-based activity replicates the actual environment (e.g. simulator, equipment, environment etc.) (Physical fidelity) | **Item (11.1)** | **2** | **4** | **4** | **4** | **4** | **0.80** |
|  | 11.2 Elements of the scenario case are related to the scenario flow (e.g. vital signed are similar to the patient diagnosis) (conceptual fidelity) | **Item (11.2)** | **4** | **4** | **3** | **4** | **4** | **1.00** |
| **12. Debriefing** | 12.1 Well known debriefing method is identified to cover the objectives of the simulation session. | **Item (12.1)** | **4** | **4** | **4** | **4** | **3** | **1.00** |
|  | 12.2 Debriefer experience stated and compatible with the skills level required to implement the debriefing method. | **Item (12.2)** | **4** | **3** | **4** | **4** | **3** | **1.00** |
|  | 12.3 Debriefing site is stated and is appropriate for the scenario. | **Item (12.3)** | **4** | **3** | **4** | **4** | **3** | **1.00** |
|  | 12.4 Debriefing time is sufficient to conduct a comprehensive session. | **Item (12.4)** | **4** | **1** | **4** | **4** | **3** | **0.80** |
| **13. Assessment** | 13.1 Assessment tool cover all of the scenario’s learning objectives. | **Item (13.1)** | **4** | **2** | **3** | **4** | **4** | **0.80** |
|  | 13.2 The assessment tool items are measurable and observable. | **Item (13.2)** | **1** | **4** | **3** | **4** | **4** | **1.00** |
|  | 13.3 All targeted critical actions, and/or skills, procedures are addressed in the assessment tool. | **Item (13.3)** | **3** | **4** | **3** | **4** | **4** | **1.00** |
|  | 13.4 The scale/ marking/ grading system is clear and feasible. | **Item (13.4)** | **2** | **4** | **3** | **4** | **4** | **0.80** |
|  | 13.5 Assessor are oriented to the assessment tool. | **Item (13.5)** | **2** | **3** | **1** | **4** | **4** | **0.60** |
|  | 13.6 Assessment tool used is validated (Optional). | **Item (13.6)** | **4** | **4** | **1** | **4** | **4** | **0.80** |

**^*^Relevancy score:** highly relevant (4), relevant (3), somewhat relevant (2), not relevant (1). ^**^Item content validity index is the proportion of content experts giving the item a relevance rating of 3 (relevant) or 4 (highly relevant) (I-CVI = (agreed item)/ (number of experts)).
