# Supplementary material for: Development and validation of Simulation Scenario Quality Instrument (SSQI)
Source: BMC Med Educ. 2023 Dec 19;23:972. doi: 10.1186/s12909-023-04935-5 (PMC10731859; doi:10.1186/s12909-023-04935-5)
Supplement: Supplementary file 4 — Additional file 4: Appendix D. Factor matrix of SSQI items and Cronbach alpha score if the item was deleted. [file 12909_2023_4935_MOESM4_ESM.docx]

**Appendix D: Factor matrix of SSQI items and Cronbach alpha score if the item was deleted.**

| Scenario Element | Item | Factor loadings | Cronbach alpha if item is deleted |
| --- | --- | --- | --- |
| 1. Learning objectives | **1.1** Learning objectives are written according to SMART format^*^.  **^*^ SMART: S**pecific, **M**easurable, **A**ttainable, **R**elevant, **T**ime-bound. | 0.350 | 0.927 |
|  | **1.2** Learning objectives are written according to Bloom’s taxonomy^*^.  **^*^ Bloom’s taxonomy** is a framework consisted of six major categories: knowledge, comprehension, application, analysis, synthesis, and evaluation. Each category has its action verbs that is used in writing learning objectives. | 0.612 | 0.928 |
|  | **1.3** Required pre-reading materials provided in the scenario are related to the learning objectives. | 0.759 | 0.928 |
| 1. Target group | **2.1** Learner prerequisite knowledge and skills is stated clearly in the scenario. | 0.662 | 0.927 |
|  | **2.2** Learners’ profession is stated clearly in the scenario. | 0.703 | 0.93 |
|  | **2.3** Learners’ number is appropriate for the scenario conduction (instructor to learner ratio is based on best practice). | 0.619 | 0.928 |
|  | **2.4** Critical actions are part of the competencies required for the learner’s profession. | 0.560 | 0.931 |
| 1. Culture | **3.1** The scenario is compatible with local laws and regulation of the healthcare system. | 0.739 | 0.93 |
|  | **3.2** The scenario follows the center or site code of conduct and ethical standards. | 0.455 | 0.93 |
|  | **3.3** Patient resembles common demographic information to the local population. | 0.860 | 0.929 |
| 1. Scenario case | **4.1 The demographic information (such as age, gender and religion) are stated clearly in the scenario case:** | -0.758 | 0.929 |
|  | **4.2 The anthropometric measurements (such as weight, height and BMI) are stated clearly in the scenario case:** | 0.816 | 0.929 |
|  | **4.3** Medical history is stated clearly in the scenario case. | 0.571 | 0.927 |
|  | **4.4** Patient current health status is stated clearly in the scenario case. | 0.816 | 0.927 |
|  | **4.5** Initial physical examination findings are stated clearly in the scenario case. | 0.518 | 0.927 |
|  |  | 0.752 | 0.924 |
| 1. Scenario narrative briefing | **5.1 The briefing elements (such as psychological safety, confidentiality agreement…) have been addressed in the briefing section:** | 0.558 | 0.927 |
|  | **5.2** Briefing time stated is enough to brief the students about the briefing elements. | 0.472 | 0.926 |
| 1. Scenario complexity | **6.1** The distractors provided in the scenario flow do not negatively impact achieving learning objectives. | 0.430 | 0.927 |
|  | **6.2** The complexity of the scenario matches learner level. | 0.665 | 0.926 |
| 1. Scenario flow | **7.1** Patient parameters and/or status are aligned with the initial statues stated in the scenario case. | 0.695 | 0.926 |
|  | **7.2** Patient parameters and/or status progresses according to leaner\s actions. | 0.549 | 0.925 |
|  | **7.3** Scenario flow indicate appropriate prompting for leaners who do not progress according to the indicated time. | 0.704 | 0.925 |
|  | **7.4** The simulation flow and overall scenario outline is clear. | 0.770 | 0.925 |
|  | **7.5** The progression of scenario flow is realistic. | 0.751 | 0.926 |
|  | **7.6** Scenario flow time is adhering to center’s guidelines (if no guidelines available, scenario should not exceed 25 minutes). | 0.455 | 0.925 |
|  | **7.7** Stated learner’s actions include critical actions stated in the “Critical action” section. | 0.854 | 0.927 |
| 1. Fidelity | **8.1** The physical context of simulation-based activity replicates the actual environment (e.g., simulator, equipment, environment, Moulage etc.) ***(Physical fidelity)*** | 0.910 | 0.927 |
|  | **8.2** Required equipment and simulators were detailed. | 0.754 | 0.926 |
|  | **8.3** Elements of the scenario are related to the scenario flow (e.g., vital signed are similar to the patient diagnosis) ***(conceptual fidelity)*** | 0.470 | 0.926 |
|  | **8.4** The script provided for the SP and direction of training is clearly stated (If applicable). | 0.445 | 0.929 |
|  | **8.5** The moulage picture is related to the scenario case (If applicable). | 0.748 | 0.924 |
| 1. Debriefing | **9.1** Appropriate debriefing method is identified to cover the objectives of the simulation session. | 0.728 | 0.923 |
|  | **9.2** Debriefer experience stated and compatible with the skills level required to implement the debriefing method. | 0.734 | 0.924 |
|  | **9.3** Debriefing site is stated and is appropriate for the scenario. | 0.603 | 0.926 |
|  | **9.4** Debriefing time is sufficient to conduct a comprehensive session. | 0.898 | 0.925 |
| 1. Assessment | **10.1** Assessment tool cover all of the scenario’s learning objectives. | 0.922 | 0.925 |
|  | **10.2** The assessment tool items are measurable and observable. | 0.923 | 0.925 |
|  | **10.3** All targeted critical actions, and/or skills, procedures are addressed in the assessment tool. | 0.903 | 0.926 |
|  | **10.4** The assessment tool grading system is clear. | 0.350 | 0.923 |
|  | **10.5** Assessment tool used is validated (Optional). | 0.612 | 0.927 |
